# A Temporal, Multicity Model to Estimate the Effects of Short-Term Exposure to Ambient Air Pollution on Health

**DOI:** 10.1289/ehp.11194

**Published:** 2008-05-09

**Authors:** Hwashin Hyun Shin, David M. Stieb, Barry Jessiman, Mark S. Goldberg, Orly Brion, Jeff Brook, Tim Ramsay, Richard T. Burnett

**Affiliations:** 1 Air Health Science Division, Safe Environments Programme, Health Canada, Ottawa, Ontario, Canada; 2 R. Samuel McLaughlin Centre for Population Health Risk Assessment, Institute of Population Health, University of Ottawa, Ottawa, Ontario, Canada; 3 Biostatistics and Epidemiology Division, Health Canada, Ottawa, Ontario, Canada; 4 Department of Medicine and Division of Clinical Epidemiology, McGill University, Montreal, Quebec, Canada; 5 Processes Research, Environment Canada, Downsview, Ontario, Canada; 6 Ottawa Health Research Institute, University of Ottawa, Ottawa, Ontario, Canada

**Keywords:** air pollution, mortality, Poisson generalized additive model, public health, simulation, spatial-temporal model

## Abstract

**Background:**

Countries worldwide are expending significant resources to improve air quality partly to improve the health of their citizens. Are these societal expenditures improving public health?

**Objectives:**

We consider these issues by tracking the risk of death associated with outdoor air pollution over both space and time in Canadian cities.

**Materials and methods:**

We propose two multi-year estimators that use current plus several previous years of data to estimate current year risk. The estimators are derived from sequential time series analyses using moving time windows. To evaluate the statistical properties of the proposed methods, a simulation study with three scenarios of changing risk was conducted based on 12 Canadian cities from 1981 to 2000. Then an optimal estimator was applied to 24 of Canada’s largest cities over the 17-year period from 1984 to 2000.

**Results:**

The annual average daily concentrations of ozone appeared to be increasing over the time period, whereas those of nitrogen dioxide were decreasing. However, the proposed method returns different time trends in public health risks. Evidence for some monotonic increasing trends in the annual risks is weak for O_3_ (*p* = 0.3870) but somewhat stronger for NO_2_ (*p* = 0.1082). In particular, an increasing time trend becomes apparent when excluding year 1998, which reveals lower risk than proximal years, even though concentrations of NO_2_ were decreasing. The simulation results validate our two proposed methods, producing estimates close to the preassigned values.

**Conclusions:**

Despite decreasing ambient concentrations, public health risks related to NO_2_ appear to be increasing. Further investigations are necessary to understand why the concentrations and adverse effects of NO_2_ show opposite time trends.

Governments throughout the developed world have started to improve air quality by changing the ways their societies use and generate energy, altering industrial processes, and selectively altering emissions streams. Introduction of these new technologies and programs is expected to cost trillions of dollars worldwide, and these societal expenditures often contribute to an improvement in air quality. To what extent does improved air quality improve public health?

In Canada, an annual reporting system has been developed in which time trends in the levels of outdoor air pollution are estimated annually, with each successive reporting year based on an additional year of monitoring data (Government of Canada 2006). The U.S. National Research Council (2004) suggested that, in addition to reporting trends in outdoor concentrations of pollutants, health risks attributable to these exposures should also be monitored.

Hundreds of studies throughout the world have linked daily variations in urban air pollution with daily variations in the number of deaths within cities (Dominici et al. 2000; Stieb et al. 2002). Most countries maintain mortality records, thus providing a resource to routinely track an important aspect of adverse health risks associated with air pollution. We illustrate our approach to estimating risk over space and time with the case of the association between two pollutants, nitrogen dioxide and ground-level ozone, and nonaccidental mortality in 24 of Canada’s largest cities over the 17-year period from 1984 through 2000. We assessed statistical properties of our method using a simulation approach.

## Materials and Methods

### Spatial–temporal model for risk of air pollution

We selected the number of daily nonaccidental deaths as the response variable reflecting the adverse short-term health effects from air pollution. The association between short-term air pollution exposure and mortality is one of the most studied and best-characterized associations in air pollution epidemiology (Bell et al. 2004; Thurston and Ito 2001). Further, mortality data are available on a national basis, so we can determine the indicator for each year.

We propose two methods to modeling risk. In method A, we derive the annual estimate of risk from the time series of *L* years, including the current year of interest as well as *L* – 1 previous years. Thus, we obtain a unique estimate of risk for each calendar year. To identify changes in risk for more recent years, we weight the mortality counts so that we assign greater weight to more recent years. For this purpose, we use the tricubed function, which gives a much greater weight to nearby years, to generate appropriate weights that vary by calendar year. The tricubed function is a popular weight function used for locally weighted smoothers (Hastie and Tibshirani 1990). For example, if *L* = 10 for 1991–2000, data for 1991 will receive the smallest weight, 0.00316621, whereas data for 2000 will receive the largest weight, 0.15908596. We also update the terms for temporal trends in mortality, weather, and day of the week (dow) indicators in our model every *L*-year period. The greater the value of *L*, the smoother the temporal estimate of risk. We denote this estimation approach as our “multi-*L*-year estimate.”

In method B, we based estimates of risk on data for each year separately and determine a weighted average of these annual estimates based on the previous *L* years. Again, we employ the tricubed function to generate weights, but this time on the regression coefficients. We denote this estimator as our “smoothed *L*-year estimate.”

Both approaches employ a two-stage approach in which we estimate risk over time, *t*^,^ separately for each community, β*_i_*(*t*) (stage 1), and then estimate a common risk among all cities, μ_β_(*t*) (stage 2). We assumed the community-specific estimates to be normally distributed with a common mean and with the variance modeled by the sum of two variances: within-community variance, ν*_i_*(*t*), and between-community variance indicating the heterogeneity of risk among cities, σ_β_^2^(*t*). The estimator of the common risk among all cities, μ̂_β_(*t*), is the weighted average of the city-specific risk estimates, β̂*_i_*(*t*), where the weight is given by [σ̂_β_^2^(*t*) + ν̂*_i_*(*t*)]^−1^. Our estimator of risk for community *i*^,^ β*_i_*(*t*), is given by


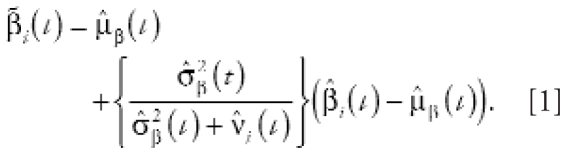


Here, β̃*_i_*(*t*) will be always closer to μ̂β(*t*) than will β̂*_i_*(*t*), so we termed β̃*_i_*(*t*) “shrinkage” estimators. If σ̂_β_^2^(*t*) = 0, then β̂*_i_*(*t*) = μ̂_β_(*t*), implying that the “best” estimate of risk for any community is the common risk estimate when there is evidence that the risks do not vary among communities. The larger the estimate of heterogeneity in risk [μ̂_β_^2^(*t*)] compared with the within-community error estimate [ν̂*_i_*(*t*)], the closer the shrinkage estimate of risk [β̂*_i_*(*t*)] to the estimate based solely on a single community’s information [β̂*_i_*(*t*)]. Although the shrinkage estimators are biased, they have smaller variances than do the community-specific estimators, thus providing more stable estimates of risk over time. We are thus borrowing strength from all the communities to estimate risk for each specific location. This is particularly useful when examining smaller communities that inherently have large uncertainties with respect to their risk estimates.

The basic concept of modeling air pollution relative to time-series mortality risk in this article is similar to that used in a previous study (Burnett et al. 2004a). However, we have developed a new estimation approach that is supported by simulation studies, and the specific context of the estimator is also somewhat different from that of our earlier work. In this article, we focus on a measure that can be “naturally” updated over time such that when an additional year of mortality and air pollution information becomes available, it can be added to the historical data set, and a new risk estimate can be obtained for that year, without altering the risk estimates from previous years. Our previous estimation approach and that of another recent effort on this topic (Dominici et al. 2007) did not specifically develop estimates to be used in this manner.

### Simulation study

We evaluated the statistical properties of our two proposed estimators of spatial–temporal risk through a simulation study. To make the simulations as realistic as possible, we used data from an analysis of air pollution and mortality in 12 Canadian cities over the 20-year period 1981–2000. In particular, we incorporated the city-specific estimates of mortality risk, measurements of air pollutant, and the confounders into our simulation and generated daily counts of mortality by adding Poisson-distributed errors city by city. In this simulation, we focus on NO_2_, a pollutant formed in the atmosphere mainly from transportation sources, with data obtained from the National Air Pollution Surveillance Network (National Air Pollution Surveillance 2001). We obtained data temperature from Environment Canada’s weather archive (Environment Canada 2002) and mortality data from the national mortality database (Vital Statistics—Death Database; Statistics Canada 2004). We coded the mortality data by census boundaries and included only deaths from internal causes [*International Classification of Diseases, 9th Revision* (ICD-9; World Health Organization 1975) codes < 800 and ICD-10 (*10th Revision*; World Health Organization 1993) codes A00–R00].

For these simulations, we used daily death counts, daily 24-hr mean temperature, and a 2-day running average of the daily 24-hr mean concentrations of NO_2_ from 1 January 1981 through 31 December 2000.

A generalized additive Poisson regression model (Dominici et al. 2002; Hastie and Tibshirani 1990) applied to the daily death counts is


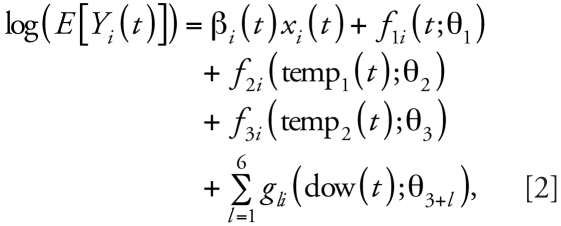


where *t*, temp_1_(*t*), temp_2_(*t*), and dow(*t*) denote calendar time, temperature recorded on the day of and 1 day before death, and the days of the week, respectively; *f*_1_*_i_* , *f*_2_*_i_* , and *f*_3_*_i_* are nonlinear smoothing functions; and *g*_1_*_i_* is an indicator function (Dominici et al. 2002). The three smoothing functions, *f*_1_, *f*_2_, and *f*_3_, describe the potential nonlinear association between time (*t*) or weather variables (temp_1_, temp_2_) and mortality, respectively. Here, *x_i_*(*t*) represents the average of concentrations of NO_2_ on the day of and day before death. We used natural cubic spline functions to estimate *f*_1_, *f*_2_, and *f*_3_, which we specified by the number of knots or degrees of freedom (df). We explored the df of the natural splines, such as df = 4, 6, 8, 10, 12 for time; df = 3, 4, 5 for temperature; and lag = 0, 1, average of (0, 1)-day lagged, and average of (0, 1, 2)-day lagged air pollution. Here we used 6 df per year for time, 3 df for temperature for the entire time period, and the average of 0- and 1-day lagged air pollutions, because we found the least variation from these parameters. [For a further discussion on natural splines and choice of df, see Ramsay et al. (2003).] We completed the model specification by assuming that the variance of the mortality counts is equal to the expected value.

### Three scenarios for the simulations

**Figure 1:**
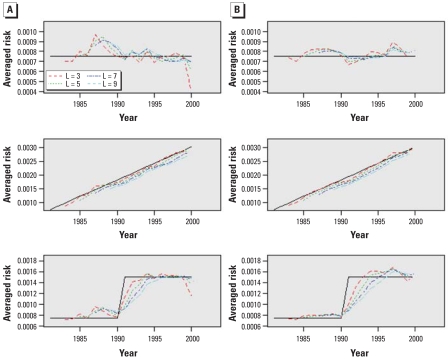
Comparison of multi-*L*-year estimator [method A (*A*)] and smoothed *L*-annual estimator (method B (*B*)] applied to simulated data for scenario 1 (top), scenario 2 (center), and scenario 3 (bottom). The solid line of each panel indicates the preassigned risk. We considered methods closer to the solid line to be better methods.

The 12 Canadian cities revealed several patterns in the secular trend in associations for NO_2_, and we considered three different scenarios, as described below. As stated above, we consider only temporal functions of risk that vary across years: constant risk over time, linearly increasing risk over time, and stepwise change in risk over time. These three scenarios are represented by solid lines in Figure 1A–C; the broken lines are results from simulated data, discussed further below. Under each scenario, we compared results from the two estimators mentioned above with preassigned values for the three scenarios.

### Simulation process

We summarize our simulation process as follows: *a*) generating daily mortality data; *b*) estimating the city-specific annual risks from the simulated data by method A (multiyear estimator) and method B (smoothed-annual estimator); *c*) estimating heterogeneity among the different cities for each year; and *d*) estimating the pooled risk for each year.

More details on each step are described in the Appendix.

## Results

### Simulation results

As described above, we used two methods to estimate pooled risks: multi-*L*-year estimator (method A) and smoothed *L*-annual estimator (method B) for L > 1. As a baseline for comparison of the two proposed methods, we also considered a single annual estimator. We estimated the mortality risks for NO_2_ from the simulated data by the proposed methods for *L* = 3, 5, 7, and 9 years and compared these with each other.

First, we compared the risk estimates from the simulated data for the two methods for all scenarios. The solid lines in Figure 1 indicate the preassigned risk over 20 years, and all the other dotted lines indicate the averages of 1,000 estimated risks for each year. Three panels on the left show results from multiyear estimator, and those on the right from smoothed-annual estimator. We used the plots to visualize the performance of the two methods (dashed and dotted lines) in capturing the given time trends. Both methods captured the preassigned trends well overall for scenario 1, except for 1987. However, the multiyear estimator that used 3 years of data, labeled *L* = 3 in Figure 1A (left), showed a considerable underestimate for year 2000. Both methods returned slightly better estimates as *L* became larger. For scenario 2, the two methods captured the linearly increasing trend but underestimated the effects because of the inclusion of previous years, where the risks were lower. Scenario 3 required more data to capture the stepwise increasing trend. After inspecting the findings, our best choice for the number of multiyears should be 5 or 7 years to avoid the apparent over- and underestimation associated with shorter or longer periods.

**Figure 2:**
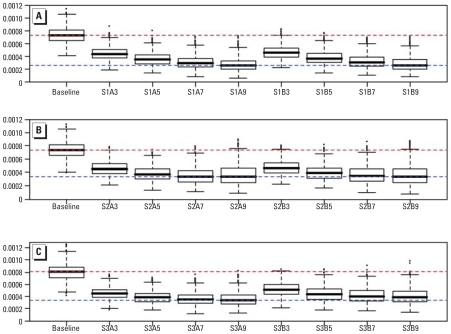
Comparison of RMSE of Canada-wide risk estimates by multi-*L*-year estimator (method A) and smoothed *L*-annual estimator (method B): box plots of RMSE distribution of 1,000 runs for scenario 1 (*A*), scenario 2 (*B*), and scenario 3 (*C*). Baseline indicates nonsmoothed annual estimator. S*m*A*n* and S*m*B*n* indicate scenario *m*, method A or method B, for *L* = *n* years. The red dashed line indicates the median of nonsmoothed annual estimator as the worst fit; blue dashed line indicates the median of multiyear estimator as the best fit. The solid horizontal line in each box indicates the median of the distribution of RMSE for each method. The box, whiskers, and dots represent the interquartile range, smallest and largest non-outliers, and outliers, respectively.

Second, we compared the root mean squared errors (RMSE) of the two methods for *L* = 3, 5, 7, and 9 (Figure 2). A smaller RMSE indicates a better fit in terms of bias and variance. Overall, the multiyear estimator and smoothed-annual estimator yielded similar results, with slightly better fit with the multiyear method for scenario 3.

Third, we compared model-based error with simulation error. The model-based error is the square root of the average of 1,000 squared estimated SEs of the pooled risk estimates, whereas the simulation error is the SD of 1,000 pooled risk estimates. Table 1 shows the results for scenario 1. As with the RMSE, the multiyear estimator provides slightly more consistent results than does the smoothed-annual estimator.

Finally, we compared the parameter variance estimates from all methods. Given that the parameter variance (heterogeneity) is known, Table 2 shows the ratios of the estimated values to the preassigned values for the parameter variance. Ratios that are closer to unity reflect better estimates with less bias, and multiyear estimator with *L* = 5 years appears to be the optimal method for all scenarios together, even if not the best for each of the three scenarios. Although method B with *L* = 5 shows a good fit for scenario 1, its estimates for the other scenarios revealed considerable underestimation.

In summary, considering both risk estimates and heterogeneity estimates, we conclude that the multi-*L*-year estimator is better than the smoothed *L*-annual estimator based on the RMSEs of the risk estimates and ratios to true values in heterogeneity estimates. Regarding the optimal value for *L*, the results for RMSE indicate *L* = 7 or 9, whereas those for parameter variance ratio indicate *L* = 5 or 7. It may depend on the time period for which data are available. For the given Canadian urban data for 20 years, choosing *L* = 7 seems to be reasonable based on the simulation results.

### Example

Daily variations in nonaccidental mortality in Canadian cities have been shown to be related to daily variations in both O_3_ and NO_2_ (Burnett et al. 2004b). We illustrate our temporal model of risk using these pollutants. We consider the daily 8-hr running maximum as the summary measure of population average exposure for O_3_ because it is the metric employed for the Canada-wide ozone standard (Canadian Council of Ministers of Environment 2000). We used the daily average concentration for NO_2_. We selected communities with a reasonably long time series of both pollutants, resulting in 24 cities having information from 1984 through 2000, the last year of nationally available mortality data. The time series models comprise natural spline terms in the model for time with 9 df/year, two natural spline terms for daily average temperature with 3 df recorded on the day of and the day before death, day of week indicator functions, and the 2-day average of pollution concentrations.

We initially considered a static- or constant-risk model for each city with β*_i_*(*t*) = β*_i_*. For O_3_, the pooled common risk is μ̂ = 7.42 × 10^−4^, which indicates the log relative rate of mortality associated with a unit (ppb) increase in O_3_, with an SE of 1.46 × 10^−4.^ Here, σ̂_β_ = 2.23 × 10^−4^, implying that 95% of cities have risks that lie in the interval (0.59 × 10^−4^ to 9.33 × 10^−4^), assuming a normal distribution. For NO_2_, the pooled common risk is μ̂ = 8.59 ×10^−4^, which denotes the log relative rate of mortality associated with a unit (parts per billion) increase in NO_2_, with an SE of 1.66 × 10^−4^. However, there is no evidence of heterogeneity of risk among cities because the estimate of the heterogeneity is zero (σ̂ = 0). Therefore, our best estimate of risk for each city is the common risk (μ̂). Based on this analysis, assuming a constant risk over time, we conclude that there is sufficient evidence to suggest that a statistical association exists between daily variation in both O_3_ and NO_2_ and nonaccidental mortality.

**Figure 3:**
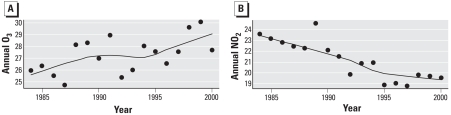
Trend in annual mortality-weighted averages of O_3_ (*A*) and NO_2_ (*B*) concentrations (ppb) from 24 Canadian cities. The curve represents time trends in concentrations smoothed by locally weighted scatterplot smoothing (LOWESS).

Figure 3 presents the annual average daily concentrations of O_3_ and NO_2_. We obtained these values by weighting the ambient concentrations by city-specific daily average mortality counts, thus reflecting population average exposure with respect to the health outcome of interest. We applied the Mann–Kendall test (Gilbert 1987), a nonparametric test for monotonic trend, to these annual averages. Concentrations of O_3_ appear to be increasing over the 17-year period, whereas those of NO_2_ are decreasing. However, the temporal pattern in NO_2_ is much clearer than that for O_3_. Our graphical interpretation of trends is supported by the Mann–Kendall test results, with stronger evidence rejecting the null hypothesis of no trend and accepting the alternative hypothesis of some monotonic trend for NO_2_ (*p* = 0.00005) than for O_3_ (*p* = 0.0435). The increasing trend in levels of O_3_ can be attributed to southern Ontario communities, which suffer from regional North American increases in O_3_, even though the O_3_ precursor pollutant, NO_2_, is declining over time (Government of Canada 2006). The time trends in nonweighted O_3_ and NO_2_ show similar Mann–Kendall test results.

**Figure 4:**
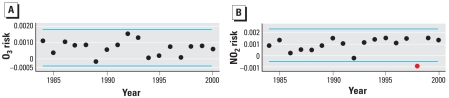
Annual pooled risk estimates for O_3_ (*A*) and NO_2_ (*B*) from 24 Canadian cities. Blue lines indicate ±2 SD of the 17 annual estimates of risk. The red circle indicates the risk outside the SD, for 1998.

Figure 4 displays the estimates of the annual pooled or common risk. Evidence for supporting the alternative hypothesis of some monotonic increasing trend in the annual risks for O_3_ is weak (*p* = 0.3870) but somewhat stronger for NO_2_ (*p* = 0.1082). Only the 1998 risk for NO_2_ (Figure 4B, red circle) is outside ±2 SD (blue lines) of the 17 annual risk estimates. To examine the sensitivity of the conclusion of the existence of a monotonic trend in annual risk, we applied the Mann–Kendall test 17 times to data sets, excluding a single year of data. For O_3_, the *p*-value varied between 0.6853 when excluding 1984 and 0.2241 when excluding 1989. There is no strong evidence to reject the null hypothesis of no increasing risks based on exclusion of any single year of data. However, *p*-values varied for NO_2_ from 0.2241 excluding 1999 to 0.0217 excluding 1998. This is consistent with the graphical information presented in Figure 4, for which the 1998 annual risk is clearly different from those in the proximal years. The other year in which risk appeared to be somewhat unusual was 1992. Excluding this year resulted in a Mann–Kendall test with a *p*-value of 0.0647, the second lowest value examined. Thus, data for 1998 and 1992 have the most influence on the statistical test for linear time trend in NO_2_.

**Figure 5:**
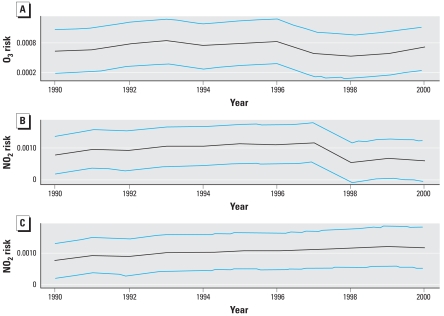
Time trend in pooled risks of O_3_ (*A*) and NO_2_ (all years, *B*; 1998 excluded, *C*) from 24 Canadian cities. Black lines indicate the pooled risk; blue lines indicate 95% confidence intervals.

We examined in detail the temporal pattern of mortality, temperature, and air pollution in each city in an attempt to identify unusual patterns that might explain the relatively low risk for NO_2_ in 1998. We observed no obvious patterns for mortality or air pollution. However, there was a clear increase in ambient temperature in southern Ontario in 1998 (data not shown). We then examined the sensitivity of the annual risk for NO_2_ to model specification. To account for this, we varied the df of the natural splines, such as df = 6, 9, 12 for time and df = 3, 6, 9 for temperature, but this did not affect the temporal pattern of risk. We examined the sensitivity of the estimates of annual risk to singular exclusion of the three largest cities, Toronto, Montreal, and Vancouver. The pattern of annual risk estimates was unaltered. Next, we divided the 24 Canadian cities into four regions. We observed a relatively low risk estimate for 1998 in the region of western Canada. The reasons for this lower risk remain unclear and subject to further investigations, such as looking into demographic changes in the western Canada region. We applied the multi-7-year method to both O_3_ and NO_2_, which we plotted over time from 1990 through 2000 (Figure 5). The temporal estimator of risk follows the general pattern of the annual estimates. Risk increased slightly for O_3_ from 1990 to 1996, followed by a slight decrease in risk for the next few years (Figure 5A). It is difficult to clearly distinguish a temporal pattern of risk different from a constant in Figure 5A. However, the temporal pattern in risk is more distinct for NO_2_ (Figure 5B), with a clear monotonic increase in risk from 1990 to 1997 and then a sharp decline in 1998. We examined the influence of the 1998 data on this pattern by removing this year and recalculating the temporal risk pattern (Figure 5C). Without 1998, a clear monotonic increase in risk is apparent.

## Discussion

Here we proposed new methods to estimate the association between daily variations in ambient air pollution and daily fluctuations in nonaccidental mortality over space and time. Spatial–temporal risk estimates, coupled with city-specific and national estimates in trends in air pollution, can be used to assess whether the adverse effect of air pollution related to mortalities has changed over time. Simulation methods show our estimator to have reasonable statistical properties for estimates of the common risk under various scenarios for changes in risk over time. However, estimates of the heterogeneity of risk among cities are unstable, with zero values frequently occurring, both in the simulation study and in the analysis of real data. In particular, estimates of heterogeneity in risk can vary considerably over time. This instability results in highly variable shrunken estimates of the city-specific risks, making it difficult to clearly identify temporal patterns. In the case of O_3_, we used the estimate of heterogeneity of risk based on all 17 years of data to determine the city-specific shrunken risk. We frequently observed zero values for the variance estimate from our temporal model of risk. This is likely attributable to the much larger within-city estimation error of risk compared with the variation in risk among cities. Alternative estimation procedures such as Bayesian methods should be considered to improve estimation of the heterogeneity.

We considered the temporal risks of NO_2_ exposure on mortality for two reasons. First, NO_2_ has been shown to be the strongest and most consistent predictor of mortality in Canadian studies (Brook et al. 2007). Second, it is not clear that NO_2_ itself is the direct causal agent; it may be acting as a surrogate for combustion in general and traffic specifically. We can address an interesting question with NO_2_: Are the Canadian government’s efforts to improve air quality by, in part, reducing NO_2_ translating into improvements in mortality risk? Based on the present analysis, the answer is no. The same issue arises with particulate matter (PM): It is not likely that mass itself is the causal agent; rather, the shape, number, or chemistry of particles may be causing the observed statistical associations between PM and mortality. Canada has historically monitored PM only every sixth day and only in a few cities. The limited sample size generates a large amount of statistical uncertainty in the risk estimates. This limits our ability to detect time trends in a meaningful way.

Risk per unit of air pollutant of interest may vary over time and space because the measured pollutant may act as a surrogate for the true toxic agent, the population or the monitoring sites may vary over time and space, or the association between exposure and death may not be linear.

### Monitored air pollutant may act as a surrogate for true toxic agent

Although statistical associations can be observed between air pollution and mortality, whether the monitored pollutant is in fact the true causal agent is not known. Several pollutants are emitted from common sources, and daily variations in concentration can be affected by weather conditions, resulting in high correlations among pollutants. For example, NO_2_ has been shown in Canadian cities to be a stronger predictor of mortality than is fine PM (Burnett et al. 2004b). However, NO_2_ has also been shown to be a stronger correlate than is fine PM with several pollutants generated from local mobile sources (Brook et al. 2007), so NO_2_ may be acting as a marker for these pollutants. The increase in the risk of NO_2_ over time (excluding 1998) is approximately the same rate as the decrease in annual average concentrations, suggesting that the attributable risk (product of risk and concentration) is stable over time. One explanation for this pattern is that NO_2_ itself may not be causally linked to mortality and that reductions in ambient concentrations are not translating into improvements in population health. The truly toxic components of the urban atmosphere may not be changing over time, at least not at the same rate as NO_2_. Daily PM measurements in Canada have not been collected historically. Size-fractionated mass and elemental concentrations have been collected in some cities since 1984 on a sampling schedule of every sixth day (Burnett et al. 2000). Speciated PM data (elements, ions, carbon) have been collected on a sampling schedule of every third day from 2003 in several Canadian communities. The temporal pattern in the correlation between NO_2_ and these PM pollutants can be examined in order to identify possible changes in the composition of the atmosphere and thus determine possible reasons for increasing NO_2_ risks.

### The population most at risk for mortality related to air pollution may change over time and vary in composition across the nation

It is well documented that the age distribution of the Canadian population has changed significantly over the last 20 years, such that there is an increasing proportion of elderly individuals at greater risk of death (Hogan 2001). The prevalence of cardiorespiratory disease has also increased (Heart and Stroke Foundation of Canada 1999). These factors could plausibly contribute to an increase in the mortality risk associated with air pollution over time.

### The adequacy of network air pollution monitoring sites as a surrogate for population average personal exposure could vary by community and time

This misclassification of exposure can lead to underestimation of risk, with the amount of underestimation depending on both space and time. This issue could be examined by correlations among monitors within each community and the use of enhanced exposure assessment methods such as land-use regression models, spatial kriging methods, and population density measures.

### The shape of the association between exposure and death may not be linear

In this simulation, we have assumed a linear association between concentration and mortality. If the association is nonlinear, then as pollution levels change over time, the number of deaths attributable to air pollution will vary depending on the level of exposure. Methods will need to be developed to incorporate nonlinear (threshold) models such as those developed by Cakmak et al. (1999).

In our study, the annual average daily concentrations of O_3_ appeared to be increasing over the 17-year period, whereas those of NO_2_ are decreasing. However, our proposed method returns different time trends in mortality risks. Evidence for some monotonic increasing time trends in the annual risks for O _3_ is weak ( *p* = 0.3870) but somewhat stronger for NO_2_ (*p* = 0.1082). In particular, an increasing time trend becomes apparent when excluding year 1998, which reveals lower risk than proximal years, even though concentrations of NO_2_ are decreasing.

Despite decreasing ambient concentrations, mortality risks related to NO_2_ appear to be increasing. Further investigations are necessary to understand why the concentrations and adverse effects of NO_2_ show opposite time trends and why year 1998 is quite different from its proximal years.

## Figures and Tables

**Table 1:** Comparison of consistency of estimates for scenario 1: multi-*L*-year estimator versus smoothed *L*-annual estimator for *L* = 3, 5, 7, and 9 years.

Method (estimator)	Model.SE^a^	Simul.SE^b^	Difference^c^	Ratio^d^
Baseline^e^	7.73 × 10^−4^	7.39 × 10^−4^	3.42 × 10^−5^	4.63 × 10^−2^
Multi-3-year	4.59 × 10^−4^	4.43 × 10^−4^	1.64 × 10^−5^	3.70 × 10^−2^
Multi-5-year	3.76 × 10^−4^	3.63 × 10^−4^	1.34 × 10^−5^	3.70 × 10^−2^
Multi-7-year	3.29 × 10^−4^	3.16 × 10^−4^	1.35 × 10^−5^	4.28 × 10^−2^
Multi-9-year	2.95 × 10^−4^	2.86 × 10^−4^	9.13 × 10^−6^	3.19 × 10^−2^
Smoothed 3-annual	4.96 × 10^−4^	4.70 × 10^−4^	2.66 × 10^−5^	5.66 × 10^−2^
Smoothed 5-annual	4.11 × 10^−4^	3.86 × 10^−4^	2.47 × 10^−5^	6.39 × 10^−2^
Smoothed 7-annual	3.60 × 10^−4^	3.37 × 10^−4^	2.31 × 10^−5^	6.86 × 10^−2^
Smoothed 9-annual	3.26 × 10^−4^	3.03 × 10^−4^	2.32 × 10^−5^	7.67 × 10^−2^

a Model.SE is the square root of the average of 1,000 squared SEs of the pooled risk estimates.

b Simul.SE is the SD of the 1,000 pooled risk estimates.

c Difference = model.SE – simul.SE.

d Ratio = (model.SE – simul.SE)/simul.SE.

e Baseline is the nonsmoothed annual estimator.

**Table 2:** Comparison of bias in heterogeneity (difference among the 12 Canadian cities) estimates for all scenarios during 12 year time period, 1989–2000.

	Ratio^a^		
Method (estimator)	Scenario 1	Scenario 2	Scenario 3
Baseline^b^	2.72	0.63	1.10
Multi-3-year	1.87	0.74	0.88
Multi-5-year	1.82	0.82^c^	0.92^c^
Multi-7-year	1.63	0.80	0.88
Multi-9-year	1.42	0.75	0.82
Smoothed 3-annual	1.29	0.58	0.65
Smoothed 5-annual	1.04^c^	0.60	0.57
Smoothed 7-annual	0.90	0.61	0.53
Smoothed 9-annual	0.83	0.60	0.51

a Ratio = (parameter variance estimate)/(preassigned value for parameter variance).

b Baseline = nonsmoothed annual estimator.

c Ratios indicate the best results; the closer to 1, the better estimate.

## Appendix

### Generating daily mortality data *(Y** *_ij_* *)*

We designed this simulation to be as close to the real situation as possible. We generated daily mortality counts to be close to the expectation of the daily death counts for each of the 12 cities for 20 years using the model in Equation 2 (model 2), which required calendar time, temperature, day of week, and air pollutant level. To conduct a reliable simulation, we incorporated the contribution of those factors (potential confounding variables) in generating daily mortality counts. We gave only the relative risk for each city *i*^,^ β*_i_* (*t*), preselected values that were close to the estimate from that city’s data.

Suppose we estimate the expectation of the daily mortality from model 2 as follows. For city *i* and year *j*,

The contribution of the potential confounding variables is

Let β*_ij_**** represent the true values and replacing the estimated risk β̂*_ij_* by the true risk β*_ij_**** ; we now have the new expectation of the daily mortality,

Adding random errors (ɛ*_ij_*) from a Poisson distribution, we generated daily deaths as

We assumed the true risks, β*_ij_**** , to be different for each year within each city. For each scenario, we determined the risk for the first year, β*_i_*_1_^*^, equal to the shrunken estimate from the Canadian 12-city data. The preassigned values for city-specific risks ranged from 0.096 × 10^−3^ to 1.677 × 10^−3^ for the estimated risks of NO_2_

### Estimating city-specific annual risks using simulated data *(*β̂*_ij_**)*

From 1,000 simulated daily mortality data sets, we obtained 1,000 estimates for the risk, β̂*_ij_*, and its sampling variance, ν̂*_ij_*, by applying model 2.

### Estimating heterogeneity among different cities for year j [σ̂_β_^2^*(j)*]

We obtained annual risk estimates for each of the 12 cities. Applying the random-effects model to these 12 city-specific risk estimates for each year, we estimated the parameter variances, σ̂_β_^2^(*j*), which indicate heterogeneity among the 12 cities.

### Estimating the pooled health risk for year j [μ̂_β_*(j)*]

For any year *j*, we obtained the pooled risk estimate by combining the 12 city-specific risk estimates:

where *K* is the number of cities and *w**_ij_* = 1 / var(β̂***ij*** ) = 1 / var(σ̂_β_^2^_R,adj_(*j*) + ν̂*_ij_* ) for city *i*.

The variance of this pooled risk estimate is
